# Signal detection of adverse events in medical devices using natural language processing: a case study in pelvic mesh

**DOI:** 10.1038/s41598-026-50950-z

**Published:** 2026-04-29

**Authors:** Thu-Lan Kelly, Ty Stanford, Curtis Murray, Lewis Mitchell, Indu Bala, Nicole Pratt, Tobin South, Renly Lim, Marianne Gillam

**Affiliations:** 1 School of Pharmacy and Biomedical Science, Quality Use of Medicines and Pharmacy Research Centre, Adelaide University, Adelaide, Australia; 2 School of Allied Health and Human Performance, Adelaide University, Adelaide, Australia; 3 School of Information and Computing Sciences, University of Melbourne, Melbourne, Australia; 4 School of Mathematical Sciences, Adelaide University, Adelaide, Australia; 5 Massachusetts Institute of Technology, Boston, MA USA; 6 Centre for Translational Research, Institute for Research, Development and Innovation, IMU University, Kuala Lumpur, Malaysia

**Keywords:** Medical device, Natural language processing, Signal detection, Disproportionality analysis, Pelvic mesh, Post-market surveillance, Computational biology and bioinformatics, Diseases, Health care, Medical research, Risk factors

## Abstract

Disproportionality analysis is used to detect safety signals for post-market surveillance from adverse events reported to regulatory bodies but is challenging when reports contain unstructured free-text. We implemented a proof-of-concept system combining natural language processing of free-text data with disproportionality analysis, using a known safety signal from pelvic mesh. Free-text reports in an Australian spontaneous adverse event report database between 2012 and 2017 were classified using topic modelling. ‘Pain’ was the most frequent clinical topic and was a known adverse event from pelvic mesh. Signal detection with three different comparators (hernia mesh, hernia and other mesh and all other devices) was performed every quarter with three disproportionality methods (Proportional Reporting Ratio, Bayesian Confidence Propagation Neural Network and maximised Sequential Probability Ratio Test). All methods adjusted the Type I error threshold for multiple looks at the data. A safety signal for pelvic mesh compared with hernia and other mesh was detected by all three methods in 3rd quarter of 2014, three years before the device was withdrawn from Australia in November 2017. Bayesian Confidence Propagation Neural Network most reliably accounted for uncertainty in the pain classification. Further investigation is required with other devices and databases to validate our proof-of-concept system.

**Supplementary Information:**

The online version contains supplementary material available at 10.1038/s41598-026-50950-z.

## Background

The effectiveness of medical interventions is typically assessed through clinical trials, but usually these are not powered to detect rare adverse events^1^. Post-market safety signal detection plays an important role in the ongoing monitoring of medical devices after market release^2–6^. Adverse events related to medical devices are reported to regulatory bodies through spontaneous reporting systems, submitted by manufacturers, clinicians and patients. In Australia, the Therapeutic Goods Administration (TGA) maintains the Database of Adverse Event Notifications (DAEN)^7,8^. Medical device reports in DAEN are submitted as unstructured free-text and are not coded using standard medical terminology, creating challenges for detecting safety signals for post-market surveillance^9–11^. Signal detection in medical devices has previously relied on manual classification of adverse events described in the reports. With maturing natural language processing (NLP) methods to classify information from free-text, there is an opportunity to improve the identification of safety signals from free-text reports^12^.

Spontaneous reports databases provide large-scale data that can be mined to uncover adverse events related to specific exposure groups, such as medical device classifications^2,13,14^. Disproportionate adverse event reporting, known as disproportionality analysis, has been successfully used to detect potential harms from medicines^15,16^ and can be applied to medical devices^2^. Because signal detection is often performed at periodic intervals, ongoing monitoring of safety signals must account for multiple looks at the data over time by adjusting signal detection thresholds as reports accumulate^17^.

To detect safety signals from medical devices, we investigated the use of NLP to classify adverse events from free-text reports, followed by disproportionality methods, using pelvic (urogynaecological) mesh as a case study. Pelvic mesh was widely used to treat women for pelvic organ prolapse and stress urinary incontinence. The TGA approved the first pelvic mesh device in 1998 and received its first adverse event report in 2006^18^. Following a safety alert from the Federal Drug Agency from the United States of America, the TGA undertook a post-market safety review of the device in 2008. The review found that the reported complication rate remained low, even though adverse events involving these devices were likely under-reported. There were very few reports until 2013, when the number started to increase. While the device provided benefits to some women, others experienced serious complications such as mesh erosions and severe chronic pain^19–21^. These safety concerns ultimately led the TGA to withdraw all pelvic mesh devices for the treatment of pelvic organ prolapse from the Australian market in November 2017^22^. The ongoing health, financial and legal consequences of the device highlight the need for more efficient post-market surveillance methods^21,23^.

### Objective

The purpose of this study was to investigate current disproportionality methods to determine whether safety signals in a post-market surveillance database of medical devices could be identified prior to device withdrawal using a proof-of-concept, NLP topic modelling pipeline on adverse event free-text to generate the underlying data. We used a case study of pelvic mesh reports within the DAEN.

## Methods

The analysis involved: (1) extracting reports on mesh and other devices in the DAEN data from the TGA website; (2) pre-processing the raw data; (3) classifying the reports into topics using NLP; (3) detecting safety signals from the most frequent adverse event linked with the topics in pelvic mesh with three disproportionality methods; and (4) comparing the signal detection date with the November 2017 withdrawal date of pelvic mesh.

### Data sources

The DAEN for medical devices is an open access database of adverse events submitted by manufacturers, patients and clinicians from July 2012 to the present^24^. For this study, we examined reports from 2012 to 2017, spanning the start of data collection in DAEN to the year the pelvic mesh devices were withdrawn from the market. The DAEN reports on the TGA website^8^ were downloaded on 18/8/2022. The website has been updated since the data download. Reports were filtered using ‘mesh’ in the Global Medical Device Nomenclature term. Data were merged with the lists of device manufacturers from 2012, 2017 and 2021^25^, and the DAEN was subsequently searched for all reports from those manufacturers. Search results were manually classified into ‘pelvic mesh’, ‘hernia mesh’ and ‘other mesh’. All other Class I-IV devices were classified as ‘other device’, with Class I representing the lowest potential for harm, to Class IV with the highest^26^.

### Natural language processing

The reports contained within DAEN are categorised by the TGA into ‘death’, ‘injury’, ‘non-injury’ or ‘unknown’. Further details are reported as unstructured free-text, from which adverse events must be extracted^9^. This poses challenges due to the complexity of medical terminology used in the reports by manufacturers and clinicians, while reports submitted by patients use less technical language. We used topic modelling to classify the reports into related topics^27,28^, a NLP technique which has been used on a spontaneous reports database from the United States of America^12^.

### Topic modelling

The unstructured free-text was profiled into mixtures of clinically relevant topics by hierarchical stochastic block (topic) modelling^27,28^. A ‘topic’ was defined as a cluster of words, where each word within a topic is assigned a probability, denoted by P(word| level, topic), reflecting the likelihood of the word’s occurrence within that specific topic for a given level in the hierarchy. Higher-level topics represent more general concepts than more detailed lower-level topics^24^. The document is represented as a mixture of these topics, characterised by the distribution of the probability of a topic $X$ given a document, P(topic = $X$| document)^29^.

Each report’s free-text was treated as a document containing a particular topic $X$ if the probability it contained words relating to the topic, P(topic = $X$| document), was over a certain threshold. The report was then considered to contain the adverse event relating to the topic. The threshold can be interpreted as the proportion of free-text in the report related to the topic. The choice of threshold balances a level high enough to minimise false-positives, as well as a threshold low enough that false negatives do not lead to insufficient events to power the disproportionality statistics. We used thresholds between 0.5% to 10% of the free-text, i.e. P(topic = $X$| document) = 0.005, 0.010,…, 0.100.

Data pre-processing and topic modelling were performed using python software (Python Software Foundation) with the hSBM_Topicmodel toolkit^30^.

### Signal detection

Disproportionality analysis aims to detect adverse events that occur at a higher rate for a particular device or class of devices compared to an appropriate comparator. Topics created by NLP were assessed manually for clinical relevance and the most frequent topic relating to an adverse event was selected for disproportionality analysis (Supplementary Material SM1). We used three comparators ranging from most to least similar (hernia mesh; hernia mesh and other mesh; and all other devices, including non-mesh devices).

We compared three disproportionality methods:

1. Proportional reporting ratio (PRR)^31^
2. Bayesian Confidence Propagation Neural Network Information Component (BCPNN IC) with 95% confidence intervals calculated using Markov Chain Monte Carlo sampling^32^
3. Maximized Sequential Probability Ratio Test (maxSPRT)^17^.

The null hypothesis is that there is no difference in the rate of adverse events between pelvic mesh and the comparator(s). The null hypothesis is represented by PRR = 1, or the adverse event rate in pelvic mesh equalling the comparator(s). For BCPNN, the test statistic is the log_2_ of the ratio of observed to expected adverse events, which is 0 under the null hypothesis. The maxSPRT test statistic is based on the maximized (log)-likelihood ratio statistic using the observed and expected reporting ratio under the null hypothesis. The critical value of the likelihood ratio statistic is determined by 100(1- $\alpha$)% quantile of possible likelihood ratio statistics over the follow-up period, where $\alpha$ is the Type I error.

One consideration in signal detection is when and how many times to test. When detecting signals at multiple timepoints, the chance of a false signal increases unless the total Type I error is fixed at a pre-defined level, for example $\alpha =0.05$, by using appropriate Type I error adjustments at each timepoint, known as $\alpha$-spending. In PRR and BCPNN, we accounted for multiple testing through manual adjustment of the threshold for detecting a signal at every timepoint, using the exponential spending function because of its wide use and reasonable properties^33^. maxSPRT is designed for sequential monitoring and maintains the correct overall $\alpha$ level. The critical value is based on the distribution of statistics under the null hypothesis, adjusted for repeated looks at the data over a proposed follow-up period. Quarterly timepoints provided a reasonable accumulation of events for mesh devices in DAEN at an interval feasible for large scale medical device safety monitoring, given the computational complexity of NLP and disproportionality analysis.

For all three methods, signal detection statistics were based on $2\times 2$ contingency tables for the cumulative number of reports with and without the adverse event, in pelvic mesh and the comparator at a given time-point. Signal detection was performed on DAEN at quarterly intervals over the study period commencing in 2012, if new data were accumulated in the interval. We evaluated the timing of signals detected from pelvic mesh with the three comparators separately.

A signal was flagged when (1) the lower limit of the adjusted confidence interval for the test statistic was above 1.0 for PRR, or 0.0 for BCPNN; or (2) the test statistic was above the critical value for maxSPRT. We considered there was a consistent disproportionate reporting rate of a topic from pelvic mesh if all three methods detected the signal at a particular timepoint. A more detailed description of disproportionality analysis and $\alpha$-spending is in the Supplementary Material Section SM2.

All disproportionality analysis was performed in R version 4.3.1 (R Foundation for Statistical Computing, Vienna Austria) with the assistance of the Sequential^34^ and EmpiricalCalibration^35^ packages for maxSPRT threshold calculation. Reproducible disproportionality analysis code is available at https://github.com/tystan/mesh-sig-detect/.

### Uncertainty analyses

We conducted a series of analyses to investigate the robustness of the disproportionality analysis results. The sensitivity and specificity of topic modelling of the most frequent adverse event, over topic thresholds, compared to a ‘gold standard’ clinician classification of the free-text into an adverse event or ‘other’ (not adverse event) report, was undertaken to establish the misclassification rates of the topic modelling algorithm.

Two of the authors (MG and RL) classified the mesh reports while blinded to the type of device and the topic modelling classification. All pelvic mesh reports were classified by both reviewers. A different 50% sample of hernia and other mesh reports were classified by each reviewer. Sensitivity, specificity, positive predictive value and negative predictive value were calculated for classification of the adverse event from topic modelling compared to clinician review, for pelvic, hernia and other mesh devices. Confidence intervals of these statistics were calculated using the Wilson method^36^ (Supplementary Section SM3).

Next, we assessed the performance of the signal detection methods under the effect of topic modelling misclassification of the reports into adverse event or ‘other’ (Supplementary Section SM4). Misclassification rates were simulated using beta distribution Bayesian priors that spanned the 95% confidence intervals of the estimated sensitivity and specificity of the topic modelling compared to clinician review, where these priors were specific to each pelvic mesh and hernia/other mesh reports. The final 2 × 2 contingency table at Quarter 4, 2017 was simulated 1000 times using the R package ‘episensr’ version 2.1.0^37^. From these simulated counts, the change from the observed counts was randomly imposed on the observed report counts over time. For example, if a simulation resulted in one less adverse event report that was reclassified as an ‘other’ report (because of the misclassification simulation), the simulated time-course data would use the observed time-course data with a random (equiprobable) adverse event report changed to an ‘other’ report at that same time point/quarter. From the resulting 1000 simulations, each disproportionality method was applied to the simulated cumulative report classifications to assess whether any methods were more robust to misclassification bias in the topic modelling.

Finally, to assess the risk of false positive disproportionality signals, we created a pseudo-negative control analysis where the null hypothesis should be retained by signal detection methods (Supplementary Material Section SM5). We amalgamated all pelvic, hernia and other mesh reports and randomly reassigned labels ‘mesh A’ and ‘mesh B’ at a 1:3 ratio 1000 times, then applied a misclassification simulation to each simulated dataset as above. The proportion of simulations with rejected null hypotheses by each signal detection method provided the Type I error. This analysis specifically looked at each method’s susceptibility to bias from misclassification of reports into adverse event/‘other’ when the null hypothesis should have been retained because of random allocation to mesh type A or B.

## Results

**Fig. 1:**
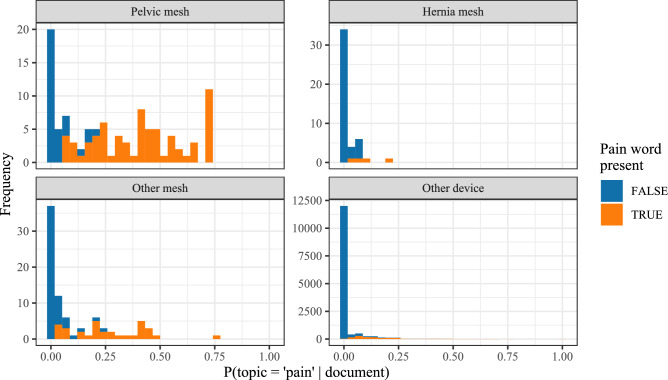
Frequency of pain topic in pelvic, hernia and other mesh and other devices using a pain topic threshold of 0.05.

A total of 14,170 reports from Class I-IV devices were extracted from DAEN, consisting of 102 pelvic mesh, 46 hernia mesh, 84 other mesh and 13,938 other devices. ‘Pain’ was the most frequent word in pelvic mesh (Supplementary Fig. S1). The five most frequent topics in pelvic mesh at the lowest, most device specific level of the hierarchy were ‘mesh’, ‘pelvic’, ‘pain, ‘bladder’ and ‘erosion’ (Supplementary Fig. S2). After clinician review, ‘pelvic’, ‘mesh’ and ‘bladder’ topics were not considered for disproportionality analysis since the topic combined potential adverse events with a description of the device and/or an indication for implantation. ‘Pain’ and ‘erosion’ were known adverse events relating to pelvic mesh^18^, and of these, ‘pain’ was the more frequent topic. Pelvic mesh had the highest proportion of reports with ‘pain’ topics, followed by other and hernia mesh (Fig. 1).

Table 1 shows the cumulative 2 × 2 contingency table for each quarter up to 2017-Q4 with hernia mesh or other mesh as potential comparators with a pain topic threshold of 0.05. The number of reports classified under the pain topic for pelvic mesh increased sharply in fourth quarter of 2014 compared with the preceding quarter.

**Table 1:** Cumulative quarterly counts of pelvic mesh compared to hernia mesh and other mesh using a pain topic threshold of 0.05. Note that non-zero counts in all cells of the 2 × 2 contingency table are required for commencement of disproportionality analysis.

Quarter	Pain reports in pelvic mesh	Other reports in pelvic mesh	Pain reports in hernia mesh	Other reports in hernia mesh	Pain reports in other mesh	Other reports in other mesh
2012-Q2	1	3	0	0	0	0
2012-Q3	1	4	0	0	0	0
2012-Q4	3	4	0	2	1	1
2013-Q1	3	7	0	5	1	1
2013-Q2	3	11	0	6	2	3
2013-Q3	4	12	1	10	2	6
2013-Q4	6	14	1	10	3	7
2014-Q1	6	14	1	11	3	8
2014-Q2	7	15	1	14	3	10
2014-Q3	9	17	3	21	4	15
2014-Q4	26	19	4	27	11	16
2015-Q1	27	19	4	28	12	18
2015-Q2	27	19	4	28	14	25
2015-Q3	27	20	4	28	14	28
2015-Q4	27	20	6	28	15	30
2016-Q1	30	21	6	28	18	33
2016-Q2	34	21	6	28	19	34
2016-Q3	34	21	7	33	20	34
2016-Q4	36	23	7	33	21	35
2017-Q1	45	23	8	34	23	39
2017-Q2	58	24	8	37	28	42
2017-Q3	68	24	8	38	32	45
2017-Q4	77	25	8	38	37	47

**Fig. 2:**
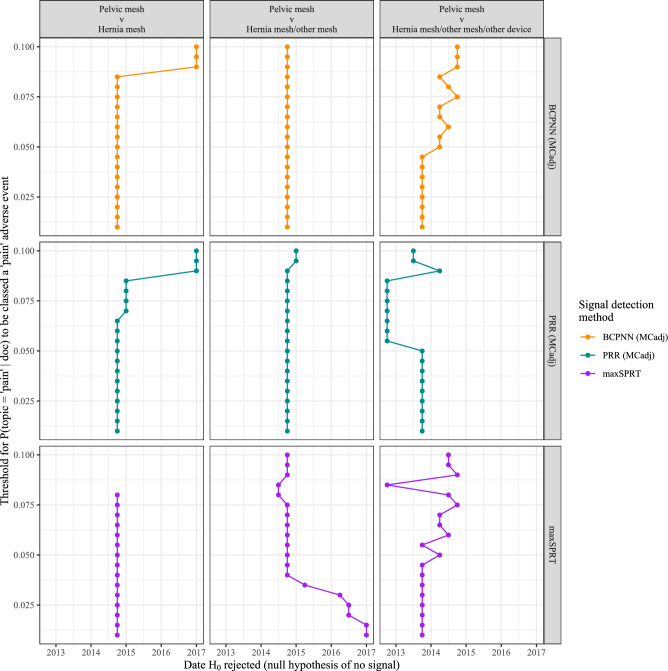
Time when three disproportionality methods with multiple comparison adjustment reached critical values for signal detection by topic probability threshold.

Figure 2 shows the year and quarter a signal was detected for the three methods and comparator devices, as a function of the topic threshold, P(topic = ’pain’| document). The optimal topic threshold in pelvic mesh for earliest signal detection in pelvic mesh for all three methods was dependent on the comparator:

- Hernia mesh: threshold < 0.07
- Hernia + other mesh: 0.04 ≤ threshold ≤ 0.075
- All other devices: threshold ≤ 0.045 (potentially unstable for thresholds ≥ 0.045)

Combining these results, the most reliable topic threshold was between 0.04 and 0.06. For all three disproportionality methods, the earliest that a safety signal was detected for pelvic mesh, compared with hernia mesh and other mesh as comparators, was 2014-Q3. Analyses using all other medical devices as the comparator showed variable and unstable results.

Figure 2 additionally shows no method was the best for detecting disproportionality signals at all topic thresholds. This may be in part to the large jump in pain reports from Q3 to Q4 in 2014, as previously noted from Table 1 at the topic threshold of 0.05, that results in a large jump in the test statistic of each method. Supplementary Fig. S3 shows the rolling disproportionality test statistics of the three signal detection methods against their critical values for the identified topic thresholds of 0.04, 0.05 and 0.06. However, no preferred method was obvious from the investigation.

For the uncertainty analyses, Table 2 shows the topic modelling sensitivity and specificity findings using gold standard clinician review. We found that a topic threshold of 0.05 for pain produced an acceptable trade-off between sensitivity, specificity, positive and negative predictive value for pelvic and other mesh for topic thresholds P(topic = ‘pain’| document) = 0.04, 0.05, 0.06 (Supplementary Fig. S4). Since there were only 4 clinician classified pain reports included in the test sample for hernia mesh, with 3 of them correctly identified by topic modelling, sensitivity and PPV for hernia mesh were lower.

**Table 2:** Sensitivity and specificity of classification of pain reports by topic modelling compared with clinician review, for thresholds of 0.04, 0.05 and 0.06. Cases refer to clinician report classification counts, and sensitivity and specificity refer to the correct classification of topic modelling for ‘pain’ and ‘not pain’, respectively.

Device type	Parameter	Threshold	Cases	Correct	Estimate (95% CI)
Pelvic Mesh	Sensitivity	0.040	70	70	1.00 (0.95, 1.00)
		0.050	70	70	1.00 (0.95, 1.00)
		0.060	70	69	0.99 (0.92, 1.00)
	Specificity	0.040	32	21	0.66 (0.48, 0.80)
		0.050	32	25	0.78 (0.61, 0.89)
		0.060	32	25	0.78 (0.61, 0.89)
Hernia Mesh	Sensitivity	0.040	4	3	0.75 (0.30, 0.95)
		0.050	4	3	0.75 (0.30, 0.95)
		0.060	4	3	0.75 (0.30, 0.95)
	Specificity	0.040	42	36	0.86 (0.72, 0.93)
		0.050	42	37	0.88 (0.75, 0.95)
		0.060	42	39	0.93 (0.81, 0.98)
Other Mesh	Sensitivity	0.040	31	31	1.00 (0.89, 1.00)
		0.050	31	28	0.90 (0.75, 0.97)
		0.060	31	27	0.87 (0.71, 0.95)
	Specificity	0.040	53	42	0.79 (0.67, 0.88)
		0.050	53	44	0.83 (0.71, 0.91)
		0.060	53	45	0.85 (0.73, 0.92)

**Fig. 3:**
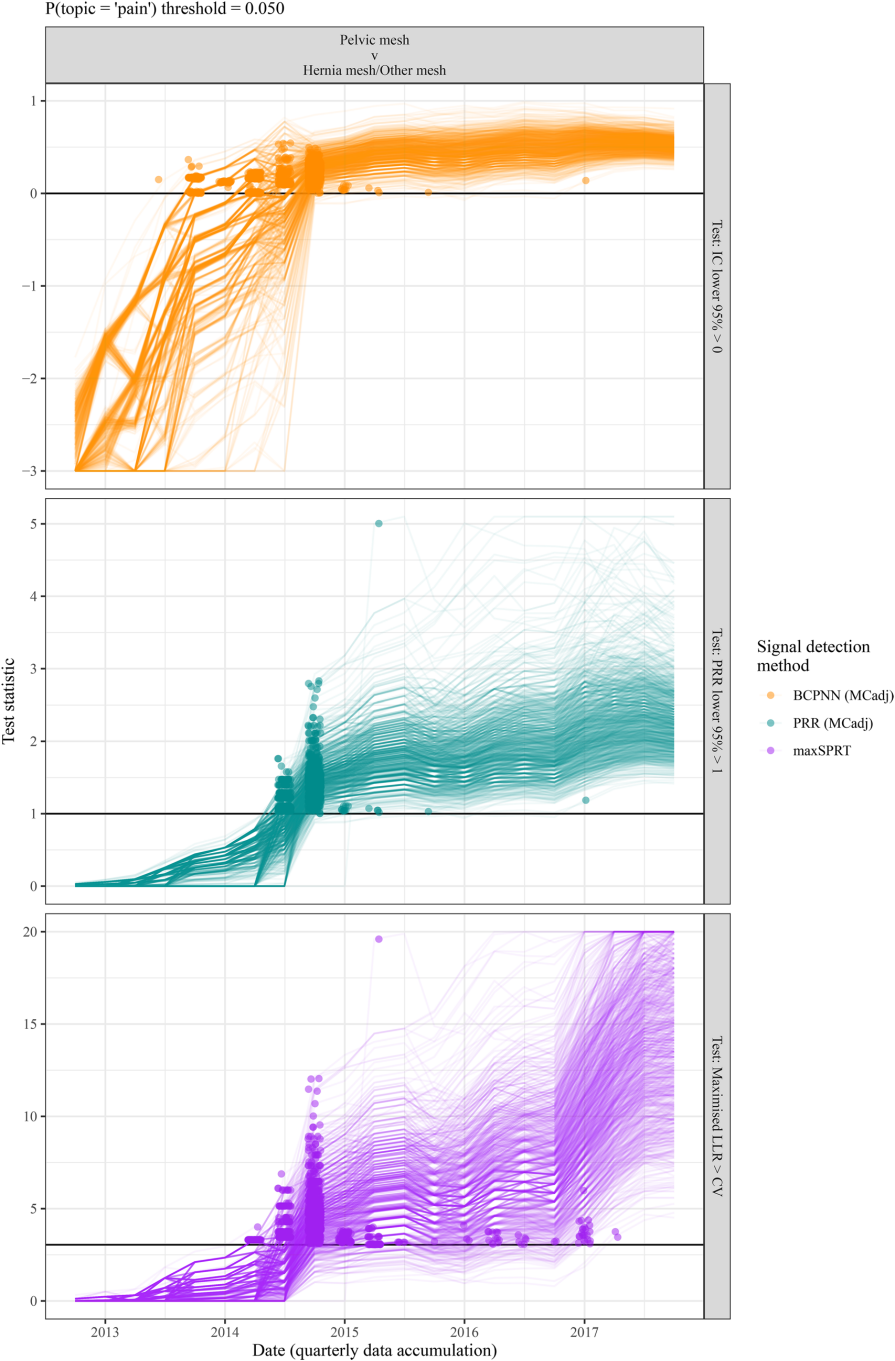
The three signal detection methods with adjustment for multiple comparisons applied to the 1000 misclassification simulated datasets tested for disproportional pain reports in pelvic vs hernia/other mesh (using a topic threshold of 0.05). Each line represents the accumulating disproportionality test statistic towards the horizontal black critical value line. The dots are jittered versions of the time each simulation achieves significance.

Figure 3 shows the signal detection methods applied to the 1000 misclassification simulated datasets testing for disproportional pain reports in pelvic vs hernia/other mesh, using a topic threshold of 0.05. Despite the adjustments to the observed data using feasible misclassification rates of pain and other reports, all of the 1000 datasets reached significance for each method. However, the timing of the detection was shown to be variable. BCPNN detected a signal before the original 2014 Q4 date in a higher proportion of the simulated data (41.8%, compared to 28.6% for PRR and 21.2% and maxSPRT) (Supplementary Material Section SM4). The maxSPRT method was more conservative in the time to detect a signal, with significance being reached 16.6% of the time after 2014 Q4 compared to less than 2% for BCPNN and PRR.

The pseudo-negative control analysis with misclassification bias demonstrated the trade-off between timely potential signal detection and false positive rates (Supplementary Section SM5). The maxSPRT method was the least prone to false positives in this simulated pseudo-negative control scenario with less than 0.5% false positives. The BCPNN method was the next best with only 3.3% false positives at the end of 2017 while PRR had 4.9%.

## Discussion

Our study demonstrated a proof-of-concept analysis pipeline combining topic modelling with disproportionality analysis to enhance post-market surveillance of medical devices. To our knowledge, there have been no other studies investigating safety signals from medical devices using this approach. The ability to extract adverse events from unstructured free-text reduces the need for manual coding and when combined with well-known methods for post-market surveillance, may reduce the timeframes required to detect safety issues with an optimised analysis pipeline and appropriate clinical review.

Our retrospective analysis using an optimal 0.05 topic threshold detected pain signals from pelvic mesh in the third quarter of 2014, three years before the device was withdrawn from the Australian market in November 2017^22^. A stable signal was achieved when hernia mesh was used as a comparator, the device most similar to pelvic mesh. Conversely, when all other devices were used as a comparator, variable time-to-disproportionate reporting rates were found irrespective of the topic thresholds. This instability likely reflects the lack of similarity in clinical use, device class, and associated risk profiles. Therefore, selecting comparator devices with similar characteristics to the device of interest is essential for meaningful signal detection.

In 2013, one year after the DAEN was created in July 2012, the number of pelvic mesh reports submitted to the TGA started to increase^18^. While the TGA was aware of adverse events before 2013, a 2008 review assessed that the complication rate remained low and no action was taken at that time. We detected a pain signal in the third quarter of 2014. It is possible that our process may have supported an earlier withdrawal date than 2017 because of systematic examination of the signal.

A key strength of this approach was the process which incorporated the combination of (1) NLP to classify adverse events from unstructured free-text; (2) $\alpha$-spending to reduce the risk of spurious signals from multiple testing over time; (3) comparison of three disproportionality methods to enhance the robustness of signal detection; and (4) determination of optimal topic thresholds and comparators. Although initially, no best disproportionality analysis method was superior, the subsequent uncertainty analysis investigation using misclassification rates to a gold standard allowed us to demonstrate $\alpha$-spending adjusted BCPNN signal surveillance was generally faster to potential signal detection without a large increase in the false positive rate when examining a relevant negative control. However, if reducing false positives is a priority, maxSPRT was the least prone to detect significant signals in the presence of report misclassification bias.

This study has several limitations. First, disproportionality analysis on spontaneous report databases has known biases, such as incomplete exposure data, selective reporting, reporting bias and inability to determine a causal relationship^38,39^. Spontaneous reports cannot be used to determine incidence, severity or causality of adverse events^8^ and signal detection is intended to detect potential adverse events which must be verified using other methods. The primary focus of our study was characterising the behaviour of disproportionality analysis using a known signal where the null hypothesis was expected to be rejected. Second, this is a feasibility study limited to a single device type in an Australian database, which may affect generalisability of the study to other devices and adverse events. Third, topic modelling was performed retrospectively on the full dataset after pelvic mesh had been withdrawn, rather than sequentially in a real-time surveillance context. Nevertheless, the large number of documents and words relating to ‘pain’ in the topics between 2012 and 2017 (Fig. 1) suggest that the results should be robust to performing topic modelling at each timepoint. When performing topic modelling sequentially, topic proportions can be estimated by maximum likelihood for new documents, given a previously-collected corpus^40^, which could be either documents up until a given timepoint; or existing reports on similar devices, for example, hernia mesh in this case study. Finally, the use of NLP to classify reports into topics does not eliminate the need for many manual steps in the process, from data cleaning, classification of devices, tuning of topic thresholds and clinical review of the topics to judge whether they relate to adverse events.

## Conclusions

We have demonstrated that NLP followed by disproportionality analysis retrospectively detected a strong, known safety signal in pelvic mesh three years prior to device withdrawal. Recent developments in NLP provide opportunities to further refine this approach. For example, using large language models to standardise less technical language from patients compared with clinicians or manufacturers. Further investigation is required to confirm our findings, such as other NLP approaches and generalisability to other adverse events, more devices and databases in different countries.

## Supplementary Information

Supplementary Information.

## Data Availability

The data supporting the conclusions of this article were extracted from the DAEN, maintained by the TGA, the Department of Health and Aged Care, Australian Government, at https://www.tga.gov.au/safety-and-shortages/database-adverse-event-notifications-daen. All extracted data and code are available at the following github repositories: Data extraction: https://github.com/tobinsouth/MedicalDeviceNLP. Topic modelling: https://github.com/curtis-murray/MedicalDevicesNLP/. Disproportionality analysis: https://github.com/tystan/mesh-sig-detect

## Abbreviations

TGA: Therapeutic Goods Administration
DAEN: Database of adverse event notifications
NLP: Natural language processing
PRR: Proportional reporting ratio
BCPNN: Bayesian Confidence Propagation Neural Network
IC: Information component
maxSPRT: Maximised sequential probability ratio test
