# Supplementary material for: Signal detection of adverse events in medical devices using natural language processing: a case study in pelvic mesh
Source: Sci Rep. 2026 Apr 29;16:20005. doi: 10.1038/s41598-026-50950-z (PMC13319759; doi:10.1038/s41598-026-50950-z)
Supplement: Supplementary file 1 — Supplementary Information. [file 41598_2026_50950_MOESM1_ESM.pdf]

Supplementary Material

Section SM1: Topic Modelling

Topics at the lowest level of the hierarchy were assessed for potential adverse events by a clinician (MG). Topics which contained a mixture of adverse events and the reason for implant or a description of the device or surgery were rejected for signal detection. The 5 most frequent topics for pelvic mesh were ‘mesh’, ‘pelvic’, ‘pain’, ‘bladder’ and ‘erosion’ (Figure S2). Of these, only ‘pain’ and ‘erosion’ were considered adverse events. These were also the adverse events reported by the TGA in their 2014 review[1]. ‘Infections’ was the 6th most frequent topic, which is also an adverse event.

Supplementary Figure S1: Word cloud of top 100 words from spontaneous reports for pelvic mesh between 2012-2017.

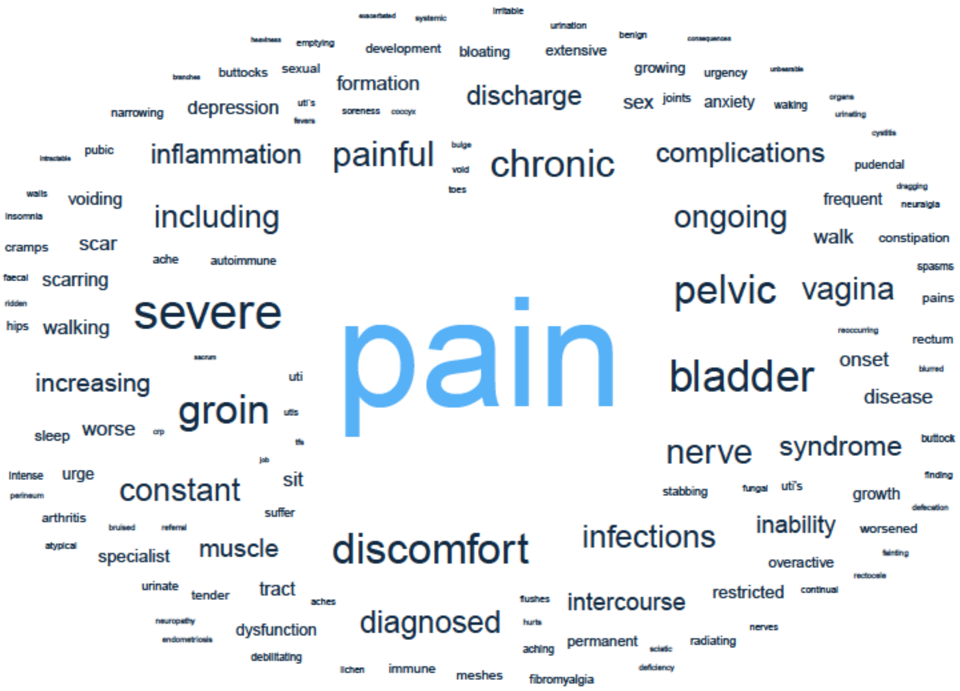

Supplementary Figure S2: Word cloud of top 100 words and topic density over time for the top five topics at the lowest level in pelvic mesh.

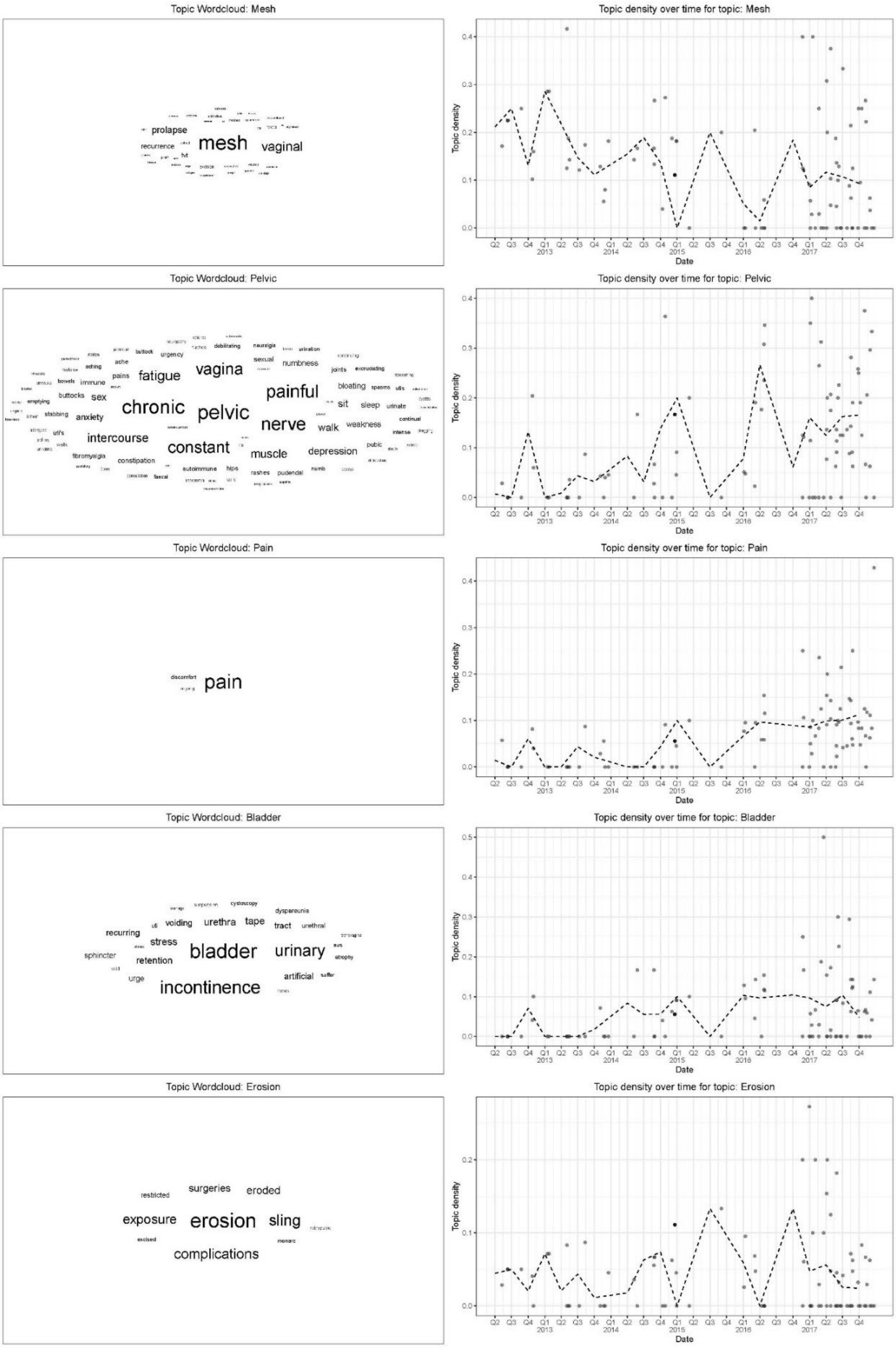

Section SM2: Signal detection

Signal detection of disproportionate adverse events (AEs) will often have tabulated count data accumulated over time. The data at time point t can be summarised as below:

	AE(s) $\in Y$	AE(s) $\in \bar{Y}$
Target exposure	a_t	b_t
Comparator exposure	c_t	d_t

where

- AE(s) Y is the set of AEs (or singular AE) of interest,
- AE(s) $\bar{Y}$ is the complementary set to the AEs of interest,
- *Target exposure* is the medical device(s) of interest,

- *Comparator exposure* is the medical devices to which the *Target exposure* is being compared, and
- a_t, b_t, c_t and d_t (all $\in \mathbb{Z}^+$) are the respective counts of AEs recorded up until (i.e., cumulative) time t.

In the motivating example of the pelvic mesh device, the contingency table can be written more specifically as

	Pain AEs	Not pain AEs
Pelvic mesh	a_t	b_t
Comparator exposure	c_t	d_t

where

- *AEs pain* is the count of AEs that contain 'pain' themes greater or equal to some pre-specified threshold $p_t \in (0,1)$ as estimated by the hSBM (that is, $P(\text{topic} == \text{'pain'}|\text{Level, Doc}) \geq p_t$), and
- *Comparator exposure* can be any relevant set of medical devices to compare the pelvic mesh to (e.g., hernia mesh or all other mesh devices or all other devices).

Detection over time

We will consider the three signal detection statistics below:

- Proportional reporting ratio (PRR),
- Bayesian Confidence Propagation Neural Network Information Component (BCPNN IC with MCMC CIs), and
- the maxSPRT statistic

As signal detection is being undertaken repeatedly as data are being accumulated, α spending needs to be considered. The below table classifies the aforementioned signal detection methods by their null hypothesis as well as how they control for the family-wise error rate (FWER).

Method	Null hypothesis	FWER adjustment
PRR	Ratio of pain AEs to all AEs in target and comparator groups has a ratio of 1	PRR with α-spending scheme
BCPNN IC	Independence of pain AEs and target group (based on marginal counts)	IC with α-spending scheme
maxSPRT (binary, group sequential)	Log-likelihood ratio test statistic below null derived threshold	(built in)

Our application of the PRR and BCPNN methods accounted for multiple testing through manual adjustment of the threshold for detecting a significant signal at every timepoint through α-spending. We chose the exponential spending function with ν=1/2 because of its wide use and reasonable properties, less α-spending for earlier looks at the data when it is assumed the power to detect differences lower.

We will demonstrate how the group sequential binary maxSPRT, as described in previous work, is equivalent to a natively FWER-controlled PRR method of signal detection using the log-likelihood ratio test for significance.

Methods used included the Bayesian Confidence Propagation Neural Network (BCPNN) and the maximised Sequential Probability Ratio Test (maxSPRT) which accounted for multiple testing through alpha spending. The BCPNN was used with and without adjusting for multiple testing. The test statistic for BCPNN is the information criterion (IC) which represents the $\log_2$ of the ratio of observed to expected adverse events (0 under the null hypothesis of no association between the topic and pelvic mesh).

maxSPRT was developed for near continuous sequential monitoring (called ‘group sequential’ when monitored at discrete time points or after set accumulations of events),

maintaining the correct overall alpha level. The test statistic is based on the maximized ($\log_2$) likelihood ratio statistic which uses the observed and expected (under the null hypothesis) reporting ratio assuming binomial adverse event accumulation. The critical value of the likelihood ratio statistic is determined by the 100(1-α)% quantile of possible likelihood ratio statistics from binomial adverse event counts under the null hypothesis over the entire group sequential follow-up.

Proportional reporting ratio (PRR)

The PRR estimate is calculated

$$\widehat{\mathrm{PRR}}_t = \frac{\frac{a_t}{a_t + b_t}}{\frac{c_t}{c_t + d_t}}.$$

In the context of signal detection, an elevated proportional reporting ratio is of concern. Therefore the one-sided hypothesis test H_0:PRR ≤ 1 (proportional reporting of the target is less than the comparator) is used and is not rejected until

$$\widehat{\mathrm{PRR}}_t \times \exp\left\{-Z_\alpha^* \sqrt{\frac{1}{a_t} + \frac{1}{a_t + b_t} + \frac{1}{c_t} + \frac{1}{c_t + d_t}}\right\} > 1$$

at the α level where Z_α^* is the $(1-\alpha)^{\text{th}}$ quantile of the standard normal distribution. The above threshold is equivalent to the lower bound of the approximate $100(1-2\alpha)\%$ confidence interval for a standard two-sided hypothesis test.

Bayesian Confidence Propagation Neural Network (BCPNN) Information Component (IC)

The Information Component (IC) statistic is an estimate of the observed-to-expected ratio of the number of target exposure AEs of interest on the $\log_2$-scale under independence between the target exposure and AEs of interest based on information theory[2]

$$\mathrm{IC}_{XY} = \log_2 \frac{P_{X,Y}(a_t + b_t, a_t + c_t)}{P_X(a_t + b_t)P_Y(a_t + c_t)}$$

where $P_X(X = x)$ denotes the marginal probability of an observed count x for the target exposure, $P_Y(Y = y)$ denotes the marginal probability of an observed count y for the AE of interest, and $P_{X,Y}(X = x, Y = y)$ denotes the joint probability.

The BCPNN IC[3] uses a Bayesian inference based *maximum a posteriori* (m.a.p.) central estimate of the IC,

$$\widehat{\mathrm{IC}}_t = \log_2 \frac{\mathrm{E}[\hat{p}_a]}{\mathrm{E}[\hat{p}_a + \hat{p}_b]\mathrm{E}[\hat{p}_a + \hat{p}_c]}$$

where p_a, p_b and p_c are the (assumed constant over time) underlying probabilities of the multinomial-distributed observed events a_t, b_t and c_t, respectively (p_d corresponding to the count d_t also included). The underlying probabilities are modelled using Dirichlet priors resulting in a Dirichlet posterior distribution. The one-sided null hypothesis of the joint probability target exposure and AEs of interest is equal or less than the marginal products (H_0: $\mathrm{IC}_t \leq 0$) can be rejected when the α quantile of the Markov Chain Monte Carlo (MCMC) empirical distribution is greater than 0. Similarly to the rejection rule for the PRR, this threshold corresponds to the lower bound of the $100(1 - 2\alpha)\%$ equal-tailed credible region in a two-sided hypothesis test.

maxSPRT

Kulldorff et al[4] outlined that the relative risk (RR) at a given point-in-time for accumulated binary data (that is, 'success'/'failure' events or AE of interest or not) of a target group relative to a comparator has the maximum likelihood estimate of

$$\widehat{\mathrm{RR}} = z\frac{C_n}{n - C_n}$$

where

- z is the ratio of the total AEs for the comparator to the total AEs for the target,
- C_n is the count of target exposure AEs in X,
- n is the count of all AEs in X (target and comparator exposure), and
- $n - C_n$ is therefore the count of comparator exposure AEs in X.

In the context of our data, the values z, C_n and n are the quantities $\frac{c_t+d_t}{a_t+b_t}$, a_t and $a_t + c_t$, respectively, at time t.

Therefore the RR maximum likelihood estimate at time t can be re-written

$$\begin{aligned}\widehat{\mathrm{RR}}_t &= \frac{c_t + d_t}{a_t + b_t} \times \frac{a_t}{c_t} \\ &= \frac{\frac{1}{a_t + b_t}}{\frac{1}{c_t + d_t}} \times \frac{a_t}{c_t} \\ &= \frac{\frac{a_t}{a_t + b_t}}{\frac{c_t}{c_t + d_t}}\end{aligned}$$

which is the PRR estimate at time t as before.

The (maximised) log-likelihood ratio statistic of $\widehat{\mathrm{PRR}}_t$ (equivalently, $\widehat{\mathrm{RR}}_t$) can be determined calculated as

$$\mathrm{LLR}_t = a_t \ln\left(\frac{a_t}{a_t + c_t}\right) + c_t \ln\left(\frac{c_t}{a_t + c_t}\right) - a_t \ln\left(\frac{a_t + b_t}{a_t + b_t + c_t + d_t}\right) - c_t \ln\left(\frac{c_t + d_t}{a_t + b_t + c_t + d_t}\right)$$

The maxSPRT test is considered significant when LLR_t is greater than the pre-computed critical value which is the $100(1-\alpha)\%$ percentile of the LLR_t values generated under the null hypothesis $\mathrm{RR}_t = 1$ for group sequential looks at the data $t = t_1, t_2, \ldots, t_k$. The CV can either be computed using the 95th percentile of LLR_t values with the exact joint binomial probabilities over k looks of the data accumulation under the null hypothesis, or by MCMC sampling of binomial event accumulation (and associated LLR_t values) to approximate the LLR distribution when the exact CV computation is computationally intractable.

Supplementary Figure S3: Comparison of rolling disproportionality test statistics against critical values for the identified topic thresholds 0.04, 0.05 and 0.06

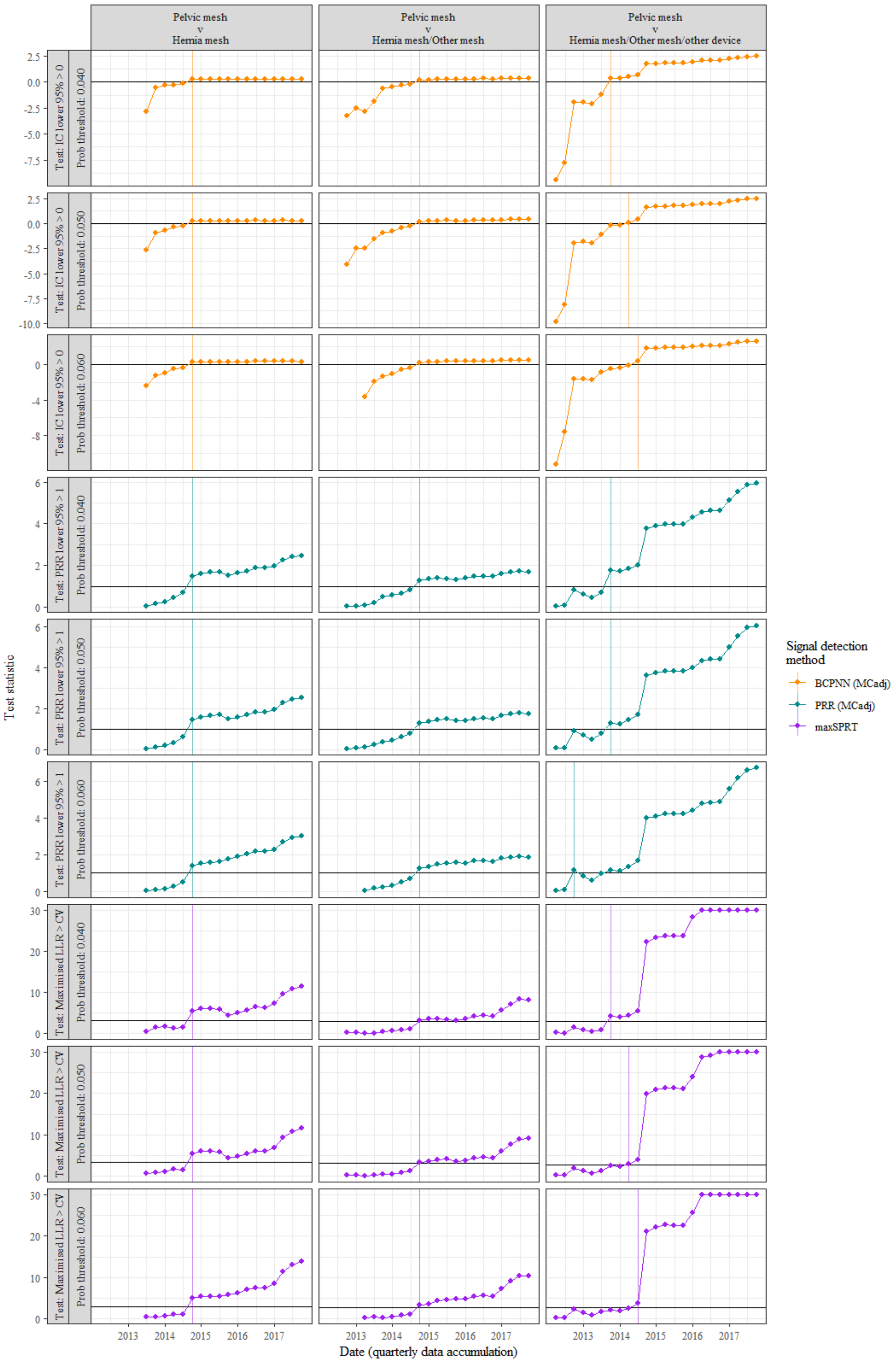

Section SM3: Topic modelling sensitivity and specificity

Classification of reports into 'pain' adverse events by topic modelling was compared to manual review by two clinicians (MG and RL). All 102 pelvic mesh reports were manually reviewed by both clinicians. The 46 hernia and 84 other mesh reports were manually reviewed separately, with 50% classified by each clinician. Manual review was considered the 'gold standard' for sensitivity and specificity calculations.

There was 100% agreement in the 'pain' classification' for the 102 pelvic mesh reports reviewed by both clinicians. Table 2 in the main manuscript shows the sensitivity and specificity of topic modelling for pelvic, hernia and other mesh and topic thresholds P(topic = 'pain'| document) = 0.04, 0.05, 0.06. Sensitivity for pelvic mesh was excellent for all thresholds, while specificity was acceptable for thresholds 0.05 and 0.06. For hernia mesh, there were only 4 cases in the samples and 3 were classified correctly by topic modelling. Specificity was good for all thresholds. In other mesh, a threshold of 0.05 was a good balance between sensitivity and specificity. Overall, for all mesh devices, a threshold of 0.05 which we used in the analysis represents an acceptable trade-off between sensitivity, specificity, positive and negative predictive value, noting that sensitivity and positive predictive value estimates for hernia mesh were affected by the small number of cases in the sample (Figure S4).

Supplementary Figure S4: Sensitivity (sens), specificity (spec), positive predictive value (ppv) and negative predictive value (npv) for topic modelling of pain from mesh devices by topic threshold.

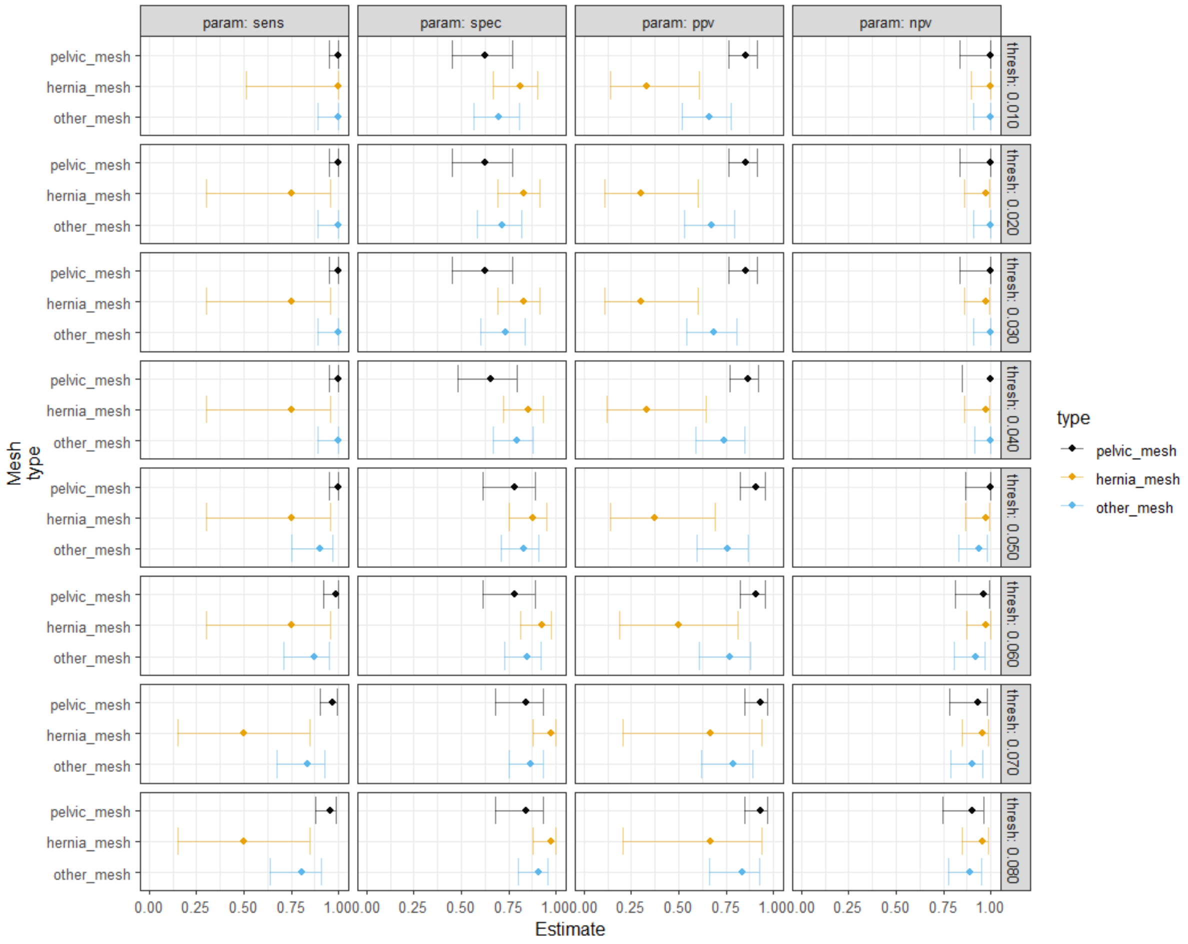

Supplementary Section SM4: Uncertainty analysis for misclassification bias

Effect of misclassification bias on the signal detection date

We used quantitative bias analysis with the ‘episensr’ R package to investigate the effect of misclassification of ’pain’ or not ‘pain’/’other’ reports when performing disproportionality analysis for hernia/other mesh as the comparator to pelvic mesh. We simulated the 4 combinations of misclassifications ‘pain’/not ‘pain’ from pelvic mesh and the comparator using the sample sensitivity and specificity confidence intervals to define reasonable ranges to sample from. Individual beta distribution priors are the natural choice allowing non-symmetric densities that lie in the range [0, 1]. The sensitivity and sensitivity for pelvic mesh and the comparator with a topic threshold of 0.05 compared with clinician review was used to create 1000 simulations that each includes misclassification of some of the reports Supplementary Figure S5 shows the 1,000 misclassification altered simulations (a line in each figure) as data accumulates for pelvic mesh vs the comparator. In each simulation, we used the accumulating 2 X 2 contingency table to determine what date significant disproportionality occurred for each of the three methods under the simulated misclassification scenario.

Supplementary Figure S6 shows the cumulative proportion of the pelvic vs hernia+other simulations with imposed misclassification rates reaching disproportionality significance. Most signal detection occurs in Q4 of 2014, consistent with the observed data. The BCPNN method had a larger proportion of significant disproportionality reached before 2014 Q4 date, i.e. a greater proportion reached significance earlier than the other two methods. Conversely, maxSPRT had the largest proportion of significance after the original identification date.

Supplementary Figure S5: Densities of the 1,000 sampled beta priors for classification sensitivity and specificity of pelvic mesh ('Exposed') and hernia+other mesh ('Not exposed') classification in the simulated datasets. In a left to right, top to bottom ordering, the specific beta distributions used were beta(100, 1), beta(81, 9), beta(60, 16) and beta(100, 211) to approximate the parameter confidence intervals (0.95, 1), (0.75, 0.97) , (0.61, 0.89), and (0.71, 0.91), respectively.

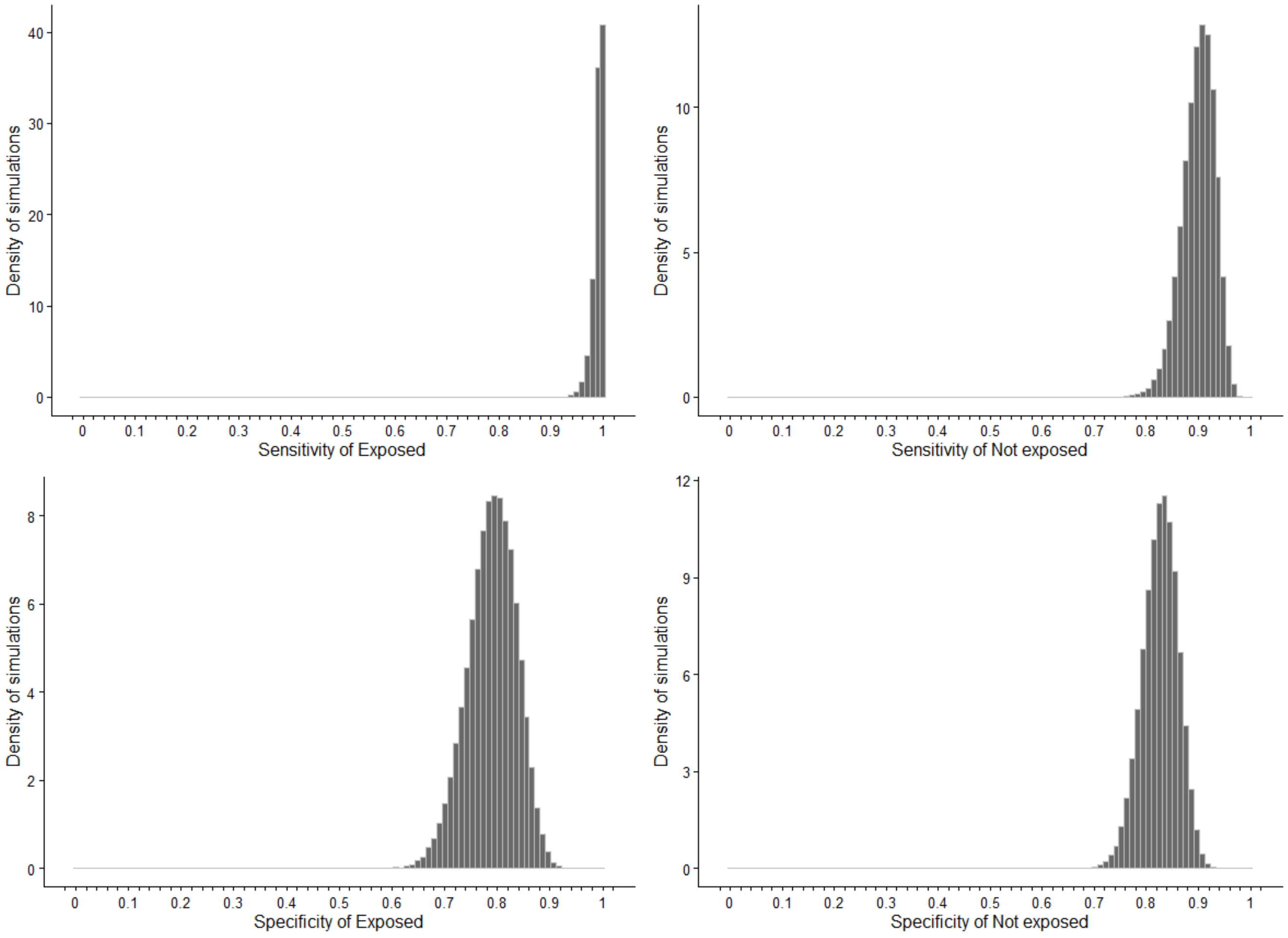

Supplementary Figure S6: Cumulative proportion of the pelvic vs hernia+other simulations (1,000) with imposed misclassification rates reaching disproportionality significance. Over 80% of simulations detected a signal in Q4 of 2014.

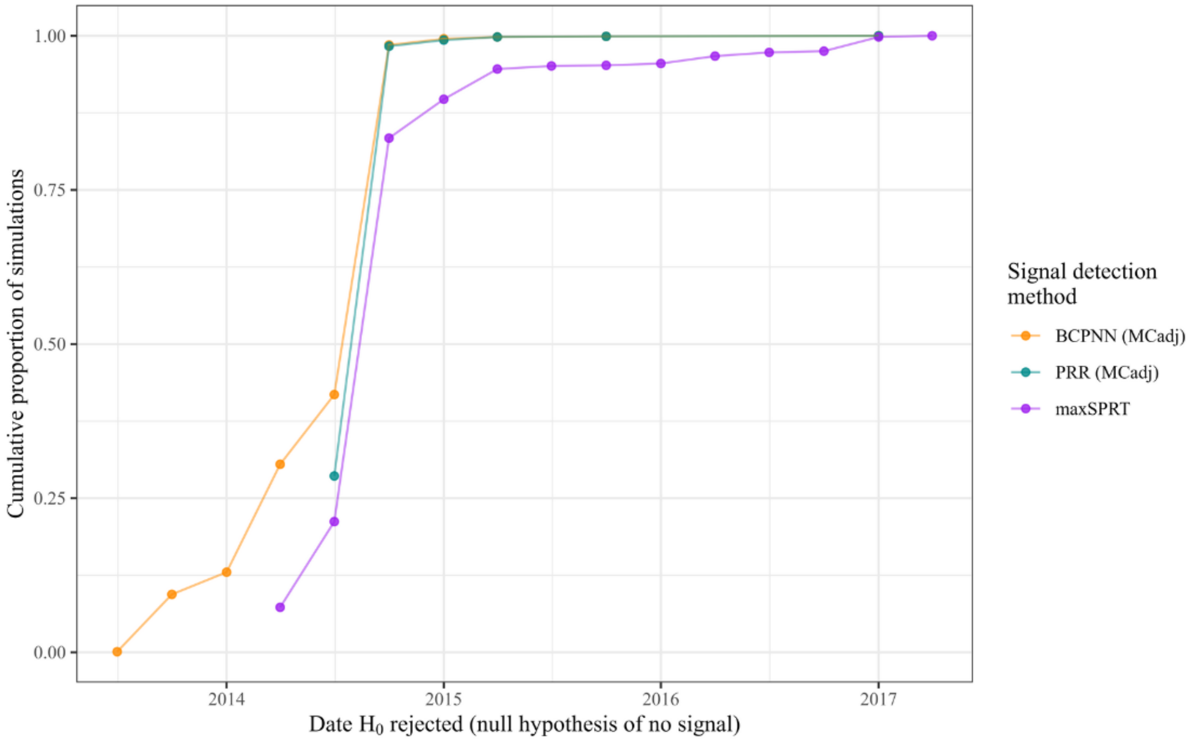

Supplementary Section SM5: Uncertainty analysis for misclassification bias on a pseudo-negative control

Supplementary Figures S7 and S8 show that maxSPRT had the least false positives (0.5%) in the pseudo-negative control scenario with 1,000 simulations. The BCPNN method was the next best with 3.3% false positives at the end of 2017, while PRR had 4.9%.

Supplementary Figure S7: The three multiple comparison adjusted signal detection methods applied to the 1,000 pseudo-negative control, misclassification bias simulated datasets, testing for disproportional pain reports between mesh A and mesh B (using a topic threshold of 0.05). Each line represents the accumulating disproportionality test statistic towards the horizontal black critical value line. The dots (jittered for visibility) represent the time a simulated dataset achieves significance.

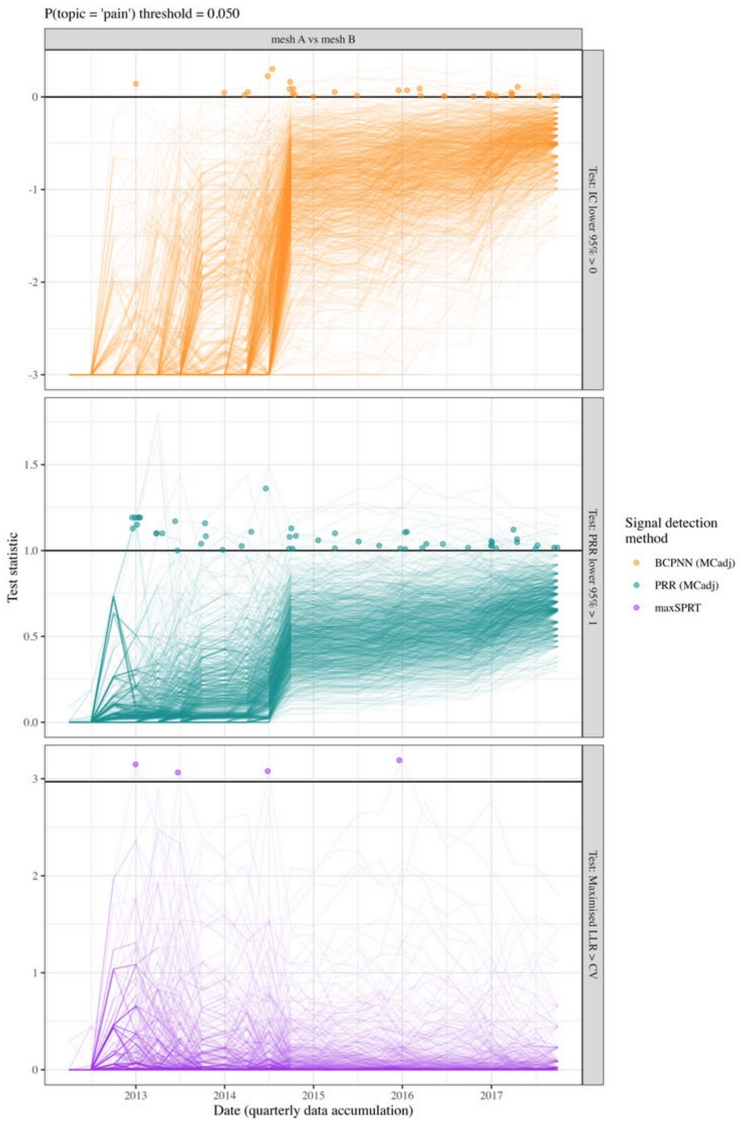

Supplementary Figure S8: Cumulative proportion of the 1,000 Mesh A vs Mesh B (pseudo-negative control analysis) simulations with imposed misclassification rates of pain and not pain reaching disproportionality significance.

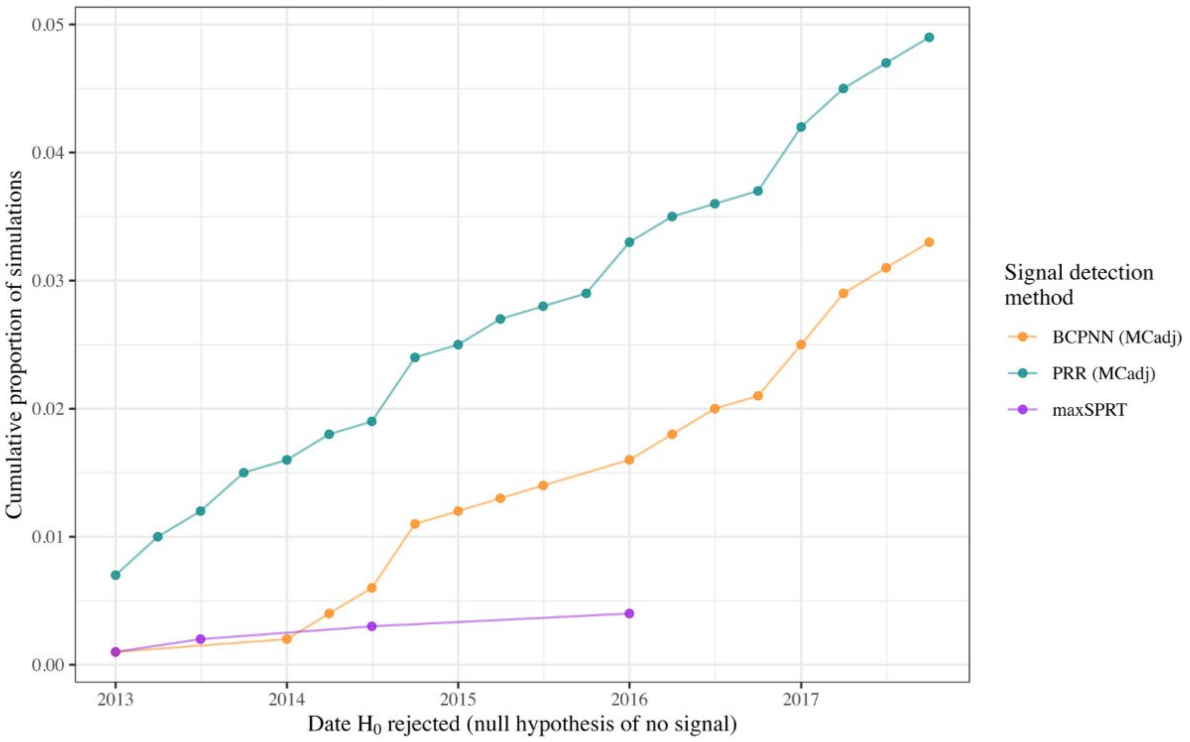

References

1 Therapeutic Goods Administration. *Results of review into urogynaecological surgical mesh implants*, <https://webarchive.nla.gov.au/awa/20180512011908/http://www.tga.gov.au/behind-news/results-review-urogynaecological-surgical-mesh-implants> (2014).

2 Bate, A. & Evans, S. J. Quantitative signal detection using spontaneous ADR reporting. *Pharmacoepidemiol Drug Saf* **18**, 427-436 (2009). https://doi.org/10.1002/pds.1742

3 Noren, G. N., Bate, A., Orre, R. & Edwards, I. R. Extending the methods used to screen the WHO drug safety database towards analysis of complex associations and improved accuracy for rare events. *Stat Med* **25**, 3740-3757 (2006). https://doi.org/10.1002/sim.2473

4 Kulldorff, M. *et al.* A Maximized Sequential Probability Ratio Test for Drug and Vaccine Safety Surveillance. *Sequential Analysis* **30**, 58-78 (2011). https://doi.org/10.1080/07474946.2011.539924
